# A decade of alkyne-tag Raman imaging (ATRI): applications in biological systems

**DOI:** 10.1039/d1cb00116g

**Published:** 2021-07-14

**Authors:** Subha Bakthavatsalam, Kosuke Dodo, Mikiko Sodeoka

**Affiliations:** Synthetic Organic Chemistry Laboratory, RIKEN Cluster for Pioneering Research Wako Saitama 351-0198 Japan dodo@riken.jp sodeoka@riken.jp; RIKEN Center for Sustainable Resource Science 2-1 Hirosawa Wako Saitama 351-0198 Japan

## Abstract

Alkyne functional groups have Raman signatures in a region (1800 cm^−1^ to 2800 cm^−1^) that is free from interference from cell components, known as the “silent region”, and alkyne signals in this region were first utilized a decade ago to visualize the nuclear localization of a thymidine analogue EdU. Since then, the strategy of Raman imaging of biological samples by using alkyne functional groups, called alkyne-tag Raman imaging (ATRI), has become widely used. This article reviews the applications of ATRI in biological samples ranging from organelles to whole animal models, and briefly discusses the prospects for this technique.

## Abstract 2

Alkyne functional groups have unique stretching frequency in the cell silent region. This review discusses the application of alkyne tags for Raman imaging in biological samples.

## Graphical abstract

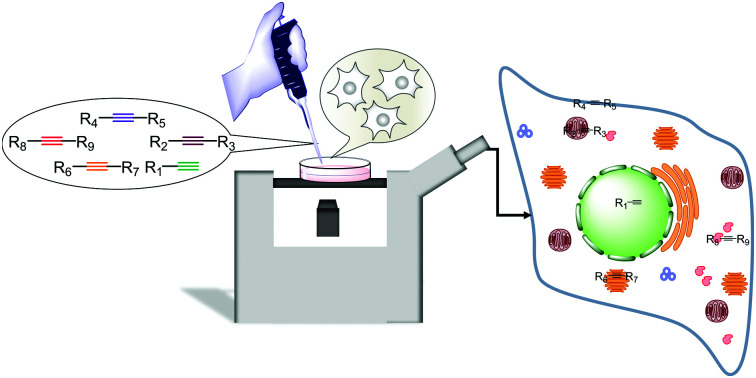

## 1. Introduction

**Fig. 1:**
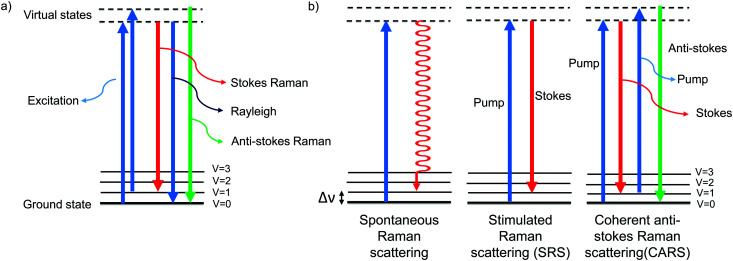
Jablonski diagram depicting the energy levels and transitions (a) during Rayleigh scattering and Raman scattering and (b) during spontaneous Raman, SRS, and CARS.

The Raman effect is an inelastic scattering of light that was first discovered by Sir C. V. Raman in 1928,^1^ for which he won a Nobel prize in 1930. In elastic scattering, called Rayleigh scattering, the molecule reverts to its original ground state after photoexcitation. In contrast, Raman scattering is an inelastic process in which the molecule returns to a higher or lower vibrational state. When the molecule returns to a higher vibrational state after scattering, the scattered light has lower energy, *i.e.*, it is red-shifted. This is called Stokes Raman shift. When the molecule returns to a lower vibrational state, resulting in blue-shifted, *i.e.*, higher-energy, scattered light, it is called anti-Stokes Raman shift (Fig. 1a). Due to the Boltzmann distribution for the vibrational energy states of molecules, the lowest vibrational state is generally the most populated state at room temperature. Hence, the Stokes Raman signal is significantly more intense than the anti-Stokes Raman signal, and is generally used for conventional Raman spectroscopy. As Raman spectroscopy advanced following the introduction of lasers and improvements in detectors and filters, it has found wide application in chemistry, physics, and biology. Recently, Raman spectroscopy has also been applied for imaging purposes, commonly called Raman imaging.^2–5^

Fluorescence microscopy is the most widely used imaging technique for the study of biological samples in an academic setting. It is also one of the most well-developed optical imaging techniques, providing images with very high spatial resolution, even sub-diffraction resolution using techniques such as PALM, STORM, STED, *etc.*^6,7^ In addition, the high temporal resolution of fluorescence microscopy has enabled it to be used for studying live cell dynamics. Though advantageous, fluorescence imaging requires labelling of the target biomolecules either with fluorescent small molecules or with genetically encoded fluorescent proteins. These non-natural tags are large enough to affect the biochemical and biophysical properties of the target biomolecules, and also affect the native cellular environment. Raman imaging has the advantage over fluorescence imaging that it can be performed without labelling or with minimal labelling. Since the Raman signal arises from molecular vibrations, the information obtained has molecular level detail. While Raman imaging has not yet achieved the spatiotemporal resolution of fluorescence imaging, different modes and methods of Raman imaging are being developed, and Raman imaging appears to be one of the leading techniques for the future. Some of the modes used for biological imaging are discussed in brief in the next section.

### 1.1 Modes of Raman imaging

#### 1.1.1 Spontaneous Raman imaging

Spontaneous Raman scattering is a process in which a molecule interacts with a single excitation photon (Fig. 1b). Mapping the signal intensity from spontaneous Raman scattering is called spontaneous Raman imaging. Though the cross section of the spontaneous process is low (10^−28^ to 10^−30^ cm^2^), it has been employed to study various cellular components simultaneously. The entire Raman spectrum of a region of interest can be obtained in spontaneous Raman imaging, which is possible only with very few coherent Raman imaging setups. Moreover, as the signal is linearly dependent on the concentration of the probe molecules, it can be used for quantitative measurements.

Confocal microscopes are employed to achieve high spatial resolution (μm order) in Raman imaging. In 1990, Puppels *et al.*, in their breakthrough work, used a confocal set-up to obtain Raman spectra of a human eosinophilic granulocyte, a chromosome of Chinese hamster lung cells, and a polytene chromosome of *Chironomus thummi thummi*.^2^ They also optimized the system for Raman imaging and imaged carotenoid C=C bonds in human lymphocytes at 1525 cm^−1^.^3^

Because of the low cross section of the spontaneous process, the image acquisition time is high. To increase the speed of image acquisition from point-scanning methods, line-scanning and slit-scanning techniques have been employed, where the samples are illuminated and imaged in a line.^8,9^ A variant of the slit-scanning technique with structured illumination has also been developed to improve spatial resolution.^10^ Recently, He *et al.* reported fast imaging by converting a low-resolution image obtained with a few scans to a higher-resolution image using artificial intelligence, trained with a large volume of imaging data.^11^ Other developments in spontaneous Raman imaging include multifocus imaging using two-dimensional array illumination,^12^ selective illumination for reduced sampling,^13,14^ and wide-field imaging.^15,16^

Resonance Raman scattering can be employed to enhance the Raman signal of molecules with a chromophore moiety. In resonance Raman, the molecules are excited using excitation source, such as light, with energy close to an electronic transition. The resulting enhancement in Raman signal could be several orders of magnitude higher than non-resonant Raman signal. Resonance Raman has been employed to visualize cells using deep-UV excitation and cytochrome *c* (cyt *c*) using 532 nm excitation.^17,18^

Methods to acquire and analyze spontaneous Raman imaging data are still evolving, and are expected to broaden its applicability in biological studies.^19–21^

#### 1.1.2 Stimulated Raman scattering, SRS

Spontaneous Raman signals are very weak and require long acquisition times. To overcome this, non-linear modes of Raman scattering, which have higher scattering cross-sections, have been employed for imaging. Non-linear Raman scattering occurs due to interaction of a molecule with multiple photons. Stimulated Raman scattering (SRS) is the most widely used non-linear Raman process. For SRS, two synchronized lasers are used as the pump laser and the Stokes laser. When the energy difference between the pump and the Stokes lasers matches the energy required for molecular vibration excitation, the molecules are coherently excited to a higher vibrational level (Fig. 1b). The signal is detected as intensity gain (Stokes) or intensity loss (pump). The Raman shift observed in SRS, *i.e.*, the frequency difference between the pump and Stokes, and the spontaneous Raman shift are the same for a molecule. Since it is a resonantly enhanced process, SRS gives much stronger signal in a shorter time compared to spontaneous Raman. Though advantageous over spontaneous Raman imaging with respect to acquisition time, SRS requires a complex ultrafast laser setup.

Xie and co-workers pioneered the Raman imaging of biological samples using non-linear modes. Using SRS microscopy, they were able to image the uptake of omega-3 fatty acid by A549 lung cells, visualize neuron bundles in mouse tissue, and monitor the delivery of retinoic acid in mouse skin.^22^ In another modification called spectrally tailored excitation SRS, the pump pulse is tailored to collectively excite all vibrational modes of the target molecules. This technique was used to image protein, oleic acid, and stearic acid in *C. elegans*.^23^

SRS microscopy has been used to visualize DNA in cells and in mouse skin, to visualize lipids in human skin, to monitor early peripheral nervous degeneration in mouse and drug delivery in fresh mouse skin, and even to detect neuronal membrane potential.^24–28^ Ozeki and coworkers demonstrated its applicability in plant cell imaging.^29^ Min and coworkers combined tissue-clearing methods with SRS to obtain a 10 times greater imaging depth than could be achieved in regular SRS imaging. This enabled 3D imaging of tumor spheroids, mouse brain tissues, and tumor xenografts. By applying volumetric phasor analysis, they were also able to extract quantitative information on the distribution of blood vessels, axon fibers, and cell bodies in various brain regions.^30^ One of the most innovative and exciting developments is label-free flow cytometry and cell sorting, achieved by integration of these techniques with SRS imaging.^31,32^

Another recent development is electronic preresonance SRS (EPR-SRS). Min and co-workers reported that by slightly detuning the excitation frequency from absorption maximum (electronic preresonance window) they could achieve higher Raman signals with lower background.^33^ This technique has huge potential in biological imaging.^34^ Advances in SRS imaging techniques and applications have been reviewed elsewhere.^35–39^

#### 1.1.3 Coherent anti-Stokes Raman scattering

Another non-linear Raman scattering technique used for imaging is coherent anti-Stokes Raman scattering, CARS. As in SRS, the molecules are coherently excited to a higher vibrational level using the pump and the Stokes lasers. Then a second pump laser is scattered by the excited molecules, returning them to the ground state. This anti-Stokes Raman is observed as blue-shifted scattered light (Fig. 1b). Since there is coherent excitation of molecules with a laser pulse, the signal is enhanced. Unlike spontaneous Raman or SRS, CARS has a non-linear dependence on concentration. One of the major advantages of CARS is that the scattered photons are at higher energy *i.e.*, shorter wavelength. Hence, CARS is not subject to interference from autofluorescence. As with SRS, CARS requires a complex setup and rapid acquisition is achieved at the cost of narrowing the spectral window. Development of multiplex CARS helps overcome the limitation of narrow spectral window.^40,41^

Xie and coworkers were the first to use CARS for biological imaging. They were able to visualize unstained Gram-negative bacteria and HeLa cells using aliphatic C–H vibrations.^42^ They have done extensive work on CARS imaging, and established this technique for imaging lipids.^43–47^

To observe the dynamics of biological processes, real-time imaging with high temporal resolution is needed. Toward this goal, Xie and co-workers reported video-rate CARS at 20 fps.^45^ Recently, Minamikawa *et al.* have developed multifocus excitation CARS, by which they could image HeLa cells at 10 fps.^48^

Most recently, super-resolution imaging was achieved with higher-order CARS, HO-CARS. CARS is generally a four-wave mixing process. By tight focusing, Gong *et al.* were able to attain up to eight-wave mixing and achieve a resolution of 196 nm. Using six-wave mixing CARS (SWM CARS), they were able to visualize HeLa Cells and buccal cells at 2845 cm^−1^ and 2935 cm^−1^, corresponding to CH_3_ stretching of proteins and lipids, respectively. SWM CARS could identify lipid droplets in the cytoplasm of HeLa cells, and observe the distribution of proteins and lipids inside the nucleus of buccal cells.^49^

There have been several excellent review articles on new developments and applications of CARS.^35,47,50^

#### 1.1.4 Surface-enhanced Raman scattering

Surface-enhanced Raman scattering (SERS) is a technique in which the Raman signal of the target molecule is enhanced when it is close to the surface of metals such as silver or gold. The enhancement is due to the presence of an electromagnetic field on the surface of these metals. Nanoparticles of these noble metals are generally used as SERS substrates. SERS can enhance the Raman signal on average by 10^4^ to 10^8^ times, and up to 10^14^–10^15^ times.^51^ Hence it can be used to monitor inherent biomolecules or the signals of small molecules attached to nanoparticles. However, to get the enhancement, the molecule of interest has to interact with the nanoparticle. Nanoparticles can also be tuned to target specific organelles or biomolecules of interest for selective visualization.

Recently, Zhang *et al.* have used Au nanorods with 4-mercaptobenzoic acid and 5,5-dithio-bis-(2-nitrobenzoic acid) as reporters to target GPR120 and CD36 proteins using antibody coating. They were able to successfully visualize and monitor the distribution changes and extracellular interaction between fatty acids and receptors by means of SERS imaging.^52^

By developing different gold nanostructures using amyloid β (Aβ) monomer and fibrils as templates, Zhou *et al.* were able to monitor the Aβ aggregation process in neurons and brain tissue in real time with ratiometric SERS imaging. The effects of Cu^2+^, Zn^2+^, and Fe^3+^ on Aβ aggregation were also studied.^53^

SERS has found wide application for detecting tumors, sensing pH, monitoring drugs and so on.^54,55^ The development and applications of SERS have been comprehensively reviewed.^55–58^

Each mode of Raman imaging has various advantages and limitations, as described in the above sections. Thus, the appropriate mode/method can be chosen according to the research target.

### 1.2 Label-free Raman imaging

**Fig. 2:**
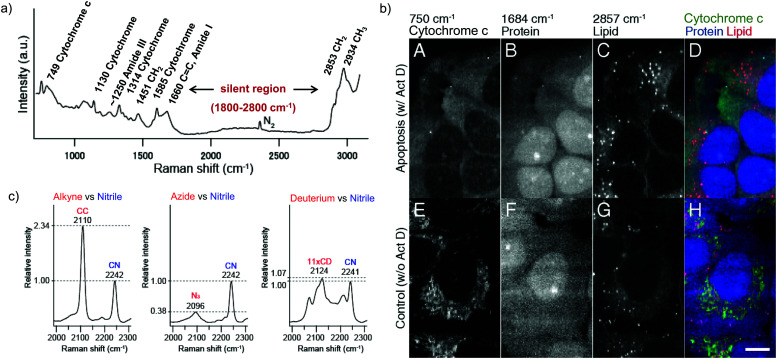
(a) Raman spectra of HeLa cells excited at 532 nm, showing the peaks derived from various cellular components. Adapted from ref. 68. Copyright 2011 American Chemical Society. (b) Label-free Raman imaging of cyt *c*, proteins, and lipids in apoptotic and non-apoptotic cells, indicating cytochrome release in apoptotic cells. Scale bar: 10 μm. Adapted from ref. 18. Copyright 2012 National Academy of Sciences (c) Raman peaks of various Raman tags in the cell silent region. Reprinted with permission from ref. 92. Copyright 2012 American Chemical Society.

Raman scattering derives information from molecular vibrations; thus, it provides a molecular level information. The inherent Raman signatures of biomolecules can be used for label-free Raman imaging (Fig. 2a). This feature has found extensive applications in biomedical imaging, especially cancer detection. Raman imaging of inherent biomolecules can assist in visualizing cells and organelles. Cytochrome-*c* (cyt *c*) is a major component of mitochondria, and hence its Raman signature can be utilized to visualize mitochondria. A pyrrole breathing mode is observed from cyt *c* at 750 cm^−1^. Upon excitation at the absorbance peak of cyt *c* at 532 nm, an enhanced Raman signal at 750 cm^−1^ is obtained due to the resonance Raman scattering effect. This was utilized to visualize the cyt *c* distribution in HeLa cells. Spontaneous Raman imaging in the slit-scanning mode was used to study the dynamics of cyt *c* with a temporal resolution of a few minutes. One of the critical steps in apoptosis, the release of cyt *c* from mitochondria to cytosol, which occurs before any morphological changes, was visualized by Raman imaging without any external labeling (Fig. 2b).^18^ More recently, the intensity change of the Raman signal of cyt *c* at 750 cm^−1^ was used to study the redox state of cytochrome and as a mitochondrial activity marker.^59^ Lipids, cellular proteins, and nucleotides have also been extensively studied/imaged by utilizing their inherent Raman signatures.^60^

The intrinsic Raman signals of drug molecules have also been used to visualize drug distributions. Biomolecules such as acetylcholine, carotenoids, retinoids, vitamin E, squalene, hemozoin, *etc.*, have been visualized by Raman imaging using their intrinsic Raman signal without any labeling.^61–66^

Recently, Sugimura *et al.* used Raman imaging at the O–H stretching band of water to measure intracellular temperature. They could also simultaneously image other cellular components along with O–H.^67^

While all molecules can be visualized by means of Raman imaging in principle, in practice it is difficult to distinguish biomolecules, since most of them have similar chemical bonds. To overcome this, Raman tags have been developed for biorthogonal labeling, as described in the next section.

### 1.3 Raman tags

Raman tags are small functional groups that have a vibrational signature in the Raman silent region of cells, from 1800 cm^−1^ to 2800 cm^−1^ (Fig. 2a). Endogenous molecules do not show any strong Raman signals in this region. Raman tags are small, consisting of very few atoms compared to fluorophores, and therefore cause much less perturbation to biological systems. Raman tags include functional groups such as alkyne, azide, and nitrile, which have high Raman cross sections, as well as isotopes such as deuterium and ^13^C, which generate shifted Raman signals compared to natural isotopes.

One of our earliest studies on imaging DNA with 5-ethynyl-2′-deoxyuridine, EdU opened the door for using such small tags in Raman imaging.^68^ Raman tags have since then been employed with various modes of Raman imaging (SRS, CARS, and SERS) to study the distribution and dynamics of biomolecules in cells.^69–71^

One of the simplest modifications that would cause the least perturbation is isotope substitution. A considerable shift has been observed in the C–H vibrations of lipids, proteins, and small molecules upon D or ^13^C substitution. C–D substituted and ^13^C substituted amino acids have been used to visualize proteome synthesis and degradation.^72^ Potma and coworkers used deuterated cholesterol (D38 cholesterol) to visualize uptake and storage in lipid droplets in Y1 adrenal cells.^73^d-Palmitate has been used to study membrane phase separation in endoplasmic reticulum. In this work, the membrane showed solid-phase behavior upon d-palmitate treatment.^74^ This cannot be studied with fluorescence imaging using bulky fluorophores, since they cannot partition into the rigid solid-phase membrane. In a recent communication, we showed that deuterated γ-linoleic acid (D-GLA) is selectively cytotoxic to tumor cells over normal cells. By Raman imaging of DC=CD vibrations at 1630 cm^−1^ we were able to show that D-GLA is mainly localized in lipid droplets in tumor cells, whereas in normal cells at a similar time point, D-GLA was distributed outside lipid droplets as well, indicating regular metabolism of D-GLA, similar to other polyunsaturated fatty acids.^75^ Deuterium-label Raman imaging thus provided mechanistic insights.

Nitrile serves as a sensitive Raman tag that can show variation in vibrational frequency upon change in the environment. By simultaneously visualizing protonated and deprotonated FCCP, Yamakoshi *et al.* demonstrated that molecular structure changes in biological systems can be monitored with appropriate Raman tags.^76^ Min and coworkers designed a palette of Raman probes called Manhattan Raman scattering (MARS) dyes by conjugating nitrile and alkyne to near-infrared fluorescent dyes. SRS imaging of the MARS probes under an electronic preresonance condition enabled them to achieve 16-color super-multiplex imaging of neuronal co-cultures.^33^ Fujioka *et al.* developed nitrile-based activatable probes to detect enzyme activity by electronic preresonance SRS. Nitrile groups were attached to xanthene derivatives bearing an enzyme-targeted functional group. Upon cleavage by the enzyme, the molecular absorption shifts to the NIR, leading to enhancement of the nitrile Raman peaks under the electronic preresonance condition. By using isotopically edited nitrile they were able to simultaneously detect four different enzymes.^34^ Inherent nitrile in molecules like anticancer drug neratinib, plant fungicides azoxystrobin and chlorothalonil have been used for imaging their distribution.^77,78^ Most recently, photoswitchable Raman probes with nitrile groups have been reported that can be turned on and off with different wavelengths of light. Such photoswitchable probes have implication for the development of super-resolution imaging.^79,80^

Modified glycans tagged with alkynes, azide, nitriles, and CD_3_ have been used for SERS imaging of the cell surface, demonstrating tunability and access to a multicolor palette for Raman imaging.^81^ Hu *et al.* employed DNA, RNA, proteins, and lipids with different Raman tags to simultaneously visualize and monitor metabolic activity in neuronal tissues.^82^

Among all the Raman tags, alkyne tags give the highest intensity signals (Fig. 2c) and hence, are the most extensively used tags for imaging. In rest of this review article, we will focus mainly on alkyne tag Raman imaging (ATRI).

## 2. Alkyne tag Raman imaging

Mapping the Raman signal intensity of alkyne tags observed in the cell silent region is called alkyne tag Raman imaging (ATRI). The alkyne moiety can be used as an exogenous tag because it has a unique Raman signature of its stretching vibration that does not overlap with any signals of biological molecules. Moreover, because an alkyne tag is sufficiently small, it is less likely to affect the homeostasis of biological systems.

Azide–alkyne click reactions have emerged as one of the powerful tools for bioconjugation, where biomolecules of interest are labelled with an alkyne moiety, which is then coupled to a fluorophore/drug containing azide, or *vice versa*.^83–85^ One of the disadvantages of this technique is that it requires toxic Cu as a catalyst or labelling with a bulky functional group such as cyclooctyne for catalyst-free reaction.^86^ On the other hand, by using ATRI, alkyne-tagged molecules can be directly used for imaging without further modification. This is advantageous since, (i) it does not involve modification with a bulky fluorophore, (ii) real-time imaging can be performed without fixation and (iii) it does not require extensive washing to remove unreacted fluorescent dyes.^68,87^ Owing to the popularity of click chemistry, many alkyne-tagged biomolecules are commercially available, and could readily be used for ATRI.

Alkyne tags are amenable to various modes of Raman imaging. The intensity of Raman scattering of alkynes is very high compared to other Raman tags, making them suitable even for spontaneous Raman imaging. For example, EdU can be detected in cells treated at concentrations as low as 5 μM with a 10 min acquisition time.^68^ Alkyne tag Raman imaging is also possible with nonlinear imaging modes such as SRS.^88^ The solution detection limit for EdU in spontaneous Raman imaging as well as SRS is 200 μM, albeit in SRS it can be detected with 100 μs acquisition time.^89^ Alkynes can bind to the surface of Au and Ag nanoparticles, making them ideal candidates for SERS imaging as well.^90,91^

**Fig. 3:**
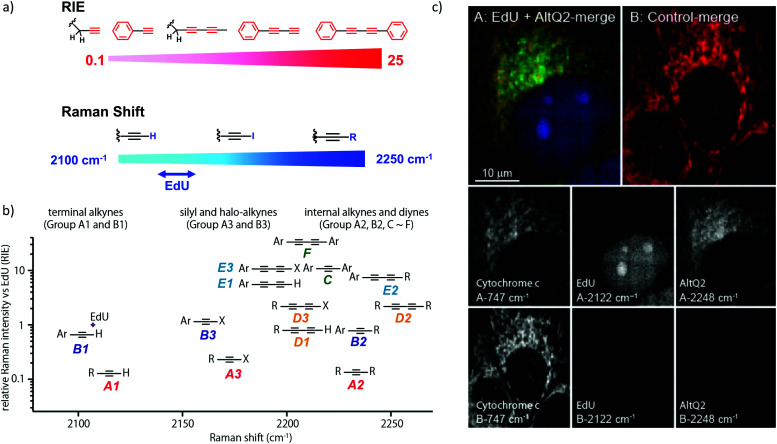
(a) Raman shift and intensity changes with changes of structure. (b) Plot of relative Raman intensity to EdU *vs.* Raman shift for various alkynes. (c) Multiplex ATRI of two alkyne-containing molecules, EdU and AltQ2, in HeLa cells along with untreated control cells. Adapted from ref. 92. Copyright 2012 American Chemical Society.

We measured the Raman shift and intensity of various alkynes and found that the position and intensity of the Raman signal vary with the structure of the alkynes (Fig. 3a). Terminal alkynes have a Raman signal at around 2100 cm^−1^, while internal alkynes show a signal at around 2200 cm^−1^ (Fig. 3b). Further, the Raman intensity is higher in more conjugated structures: conjugated di- or poly-alkynes show higher intensity than monoalkyne. Alkyne conjugated to aromatic rings also displays higher signal intensities. Bisarylbutadiyne (BADY), which has diyne conjugated to aromatic rings, shows very high intensity, ∼25 times that of EdU. Moreover, Raman signals also change with the substituents on the alkyne. Silyl alkynes and haloalkynes display a Raman signal at around 2150 cm^−1^ (Fig. 3b). This gives us a toolbox of alkynes from which to choose for optimal tagging of a biomolecule of interest.^92,93^ Since the Raman lines are sharp, multiple alkyne tags can be simultaneously imaged, thus facilitating multiplexed imaging (Fig. 3c).^92,94^

In short, the strong intensity and the tunability of Raman signals of alkyne tags makes them a popular choice to tag small molecules, biomacromolecules, and even organelles. The application of alkyne tags for ATRI is discussed in detail in the following sections.

### 2.1 Imaging of organelles

**Fig. 4:**
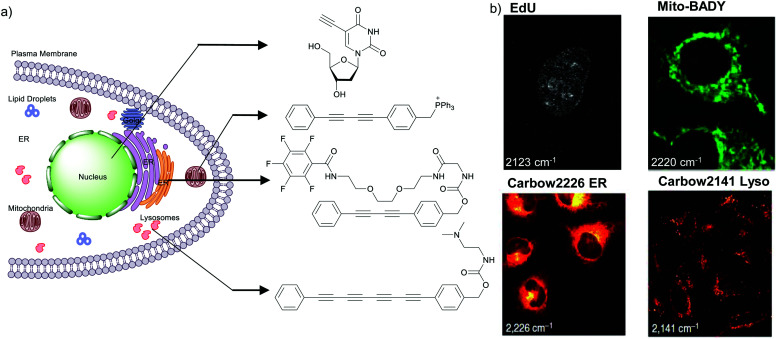
(a) Structures of some organelle markers for ATRI. From top to bottom: EdU, MitoBADY, Carbow2226 ER, Carbow2141 Lyso. (b) Raman imaging of cells loaded with mentioned organelle trackers at mentioned Raman shift. Top two panels: Spontaneous Raman imaging and bottom two: SRS imaging. Images adapted from ref. 68, 94 and 96. Copyright 2011 American Chemical Society. Copyright 2014 Elsevier Ltd. Copyright©2018, Nature Publishing Group.

The nucleus is one of the major components of the cell and contains a large volume of genetic material. It was one of the earliest cellular organelles to be stained for fluorescence imaging and is still a common target. Indeed, the nucleus was also the first organelle to be stained with alkyne and visualized with ATRI.^68^ The thymidine analogue EdU tagged with alkyne was developed to monitor cell proliferation by application of click chemistry. EdU shows an intense Raman peak at 2122 cm^−1^, which is in the cell silent region, and this enabled the nucleus to be visualized using slit-scanning spontaneous Raman microscopy at 2123 cm^−1^ (Fig. 4).^68^ Along with its use as a nuclear staining agent in Raman imaging, EdU was also employed to study the time course of cell proliferation in HeLa cells.^68^ EdU was imaged simultaneously with other alkyne-tagged molecules and endogenous cyt *c*, demonstrating the efficacy of ATRI for simultaneous multiple tag imaging.^92^ EdU was the first example of an alkyne-tagged molecule visualized by Raman imaging. As a result, the Raman intensities of most alkyne tags are quantified and reported relative to the intensity of EdU (RIE).

EdU has also been used for visualizing cell proliferation and newly synthesized DNA by SRS.^89^ By editing EdU with ^13^C isotopes, Chen *et al.* developed three different EdU alkyne tags that show three different Raman lines.^95^ Hu *et al.* used this EdU and ^13^C–^13^C modified EdU, which show Raman peaks at 2123 cm^−1^ and 2048 cm^−1^, respectively, to visualize DNA synthesis at different stages in rat hippocampal slices.^82^

Organelle staining is achieved by targeting specific biocomponents in the organelles or by targeting some characteristic feature of the organelles, such as charge, hydrophobicity, *etc.* Various molecules used for fluorescence imaging have been optimized to stain different organelles. Based on these well-studied fluorescent molecules, we have a good knowledge of the chemical moieties that can target various organelles. Triphenyl phosphonium is used to target mitochondria because of its positive charge. Bisarylbutadiyne (BADY), which displays a high-intensity Raman signal, almost 25 times that of EdU,^92^ was linked to a triphenylphosphonium pendant to obtain a mitochondrial targeting Raman tag called MitoBADY.^96^ The MitoBADY alkyne peak was observed at 2218 cm^−1^. HeLa cells treated with MitoBADY, imaged at 2220 cm^−1^, showed a clear distribution typical of mitochondria, which was confirmed by its overlap with the cyt *c* distribution imaged at 751 cm^−1^ (Fig. 4). Mitochondrial tagging was also confirmed by comparing the MitoBADY distribution with the result of fluorescence imaging of mitochondria using Mitotracker Green.^96^ This work not only established a mitochondrial marker for ATRI, but also demonstrated the feasibility of modifying aryl diynes to target various organelles if appropriate targeting moieties are attached. BADY has been used as a scaffold to synthesize organelle markers with various organelle targeting groups.^97,98^

Hu *et al.* have demonstrated that by using polyynes with distinct Raman scattering frequencies a rainbow of Raman peaks can be obtained in the cell silent region that can be used simultaneously for supermultiplexed Raman imaging. By attaching different targeting groups to polyynes they could simultaneously image mitochondria, lysosome, plasma membrane, ER, and lipid droplets.^94^ Most recently, Tian *et al.* synthesized water-soluble poly(deca-4,6-diynedioic acid) (PDDA) using a host–guest topochemical polymerization strategy. PDDA has around 10^4^ times greater intensity than EdU. The side chain of this PDDA molecule was modified with tertiary amine to target lysosomes, and with different peptides to target mitochondria and nuclei.^99^

Organelle trackers have proven extremely important and useful in fluorescence imaging. Unfortunately, fluorescent organelle trackers are difficult to use in conjunction with Raman imaging, especially in the spontaneous mode, because of the strong fluorescence background. Alkyne-tagged organelle trackers can be used as organelle markers for Raman imaging.

### 2.2 Imaging of lipids

Lipids are important biomolecules that have a gamut of functions ranging from acting as structural components to serving as signaling mediators. It is important to visualize the distribution and dynamics of these molecules to understand their roles. Fluorescent lipid mimics have been used for decades to visualize lipid distribution in membranes and the interactions of lipids with various biomolecules. However, fluorescent tags modify the properties of lipids and affect their distribution. CARS is another technique that has been used to study lipids in detail without any labeling. But label free CARS cannot differentiate lipids with similar structural components. Alkyne tags give us access to lipid mimics with only minor modification in their structure, for study with ATRI.

Weeks *et al.* have imaged 17-octadecynoic acid (17-ODYA) using CARS to study the interaction of lipids to monocytes.^69^ We demonstrated the applicability of ATRI to lipid imaging by developing analogues of CoQ, which is an essential and abundant mitochondrial lipid. We reported a series of alkyne-tagged CoQ analogues, AltQ1-16, with varying hydrophobic chain length. Using Raman intensity, the uptake of various CoQ analogs was quantified and compared in cells, throwing light on the effects of the hydrophobic chain. AltQ4 was imaged in live HeLa cells, showing that short-chain lipid analogues can be visualized with ATRI, whereas this is usually challenging with fluorescence imaging.^92^

Min and coworkers utilized 17-octadecynoic acid (17-ODYA) as a fatty acid mimic and shown that it is accumulated in lipid droplets of THP-1 macrophages and *C. elegans*.^89^ They used propargylcholine as an alkyne-tagged choline head group mimic to visualize the distribution in rat hippocampal slices. They identified a shift from 2125 cm^−1^ to 2142 cm^−1^ when the head group is incorporated into lipids.^89^ The pattern of newly synthesized phospholipids with a propargylcholine head group was also visualized.^82^ Hong *et al.* used 17-ODYA to visualize the distribution in live HeLa cells. They confirmed the distribution by fixing the cells and performing click reaction with fluorescent azide. They also reported the imaging of propargylcholine at 2138 cm^−1^ and observed its incorporation into lipid membranes.^100^

Lee *et al.* visualized cholesterol storage in CHO cells and *C. elegans* using diyne-tagged cholesterol.^101^ Yamaguchi *et al.* developed a cholesterol mimic with a functional group that is converted to alkyne with a chemical trigger in a mildly acidic environment. They used chemical activation to generate and visualize alkyne-tagged cholesterol in HeLa cells.^102^

Our group has reported sphingomyelin mimics with monoalkyne and diyne modification.^103^ Using diyne, we have visualized and quantitatively analyzed sphingomyelin distribution in artificial monolayer membranes. Sphingomyelin was observed in the central area of lipid raft-like ordered domains with a heterogeneous distribution.^104^ Such observations would be difficult to make with large fluorophore-modified lipid mimics, because they would affect the lipid organization in the membrane.

More recently, Jamieson *et al.* examined the uptake of alkyne-tagged myristic, palmitic, and stearic acids by HEK293T cells. They quantified the uptake of each of these fatty acids from the Raman intensity and verified that the uptake ratios were consistent with previous reports.^105^

So far, only a few lipid mimics with alkyne tags have been reported, and the potential of ATRI for lipid imaging is yet to be realized. One of the many advantages of ATRI is that, by using alkynes with even very small modifications in their structure, tags with different Raman signals can be obtained. Our group is currently using this strategy to visualize different lipids simultaneously in cells.

### 2.3 ATRI of biomolecules and bioactive mimics

The use of biomolecule analogues or mimics for imaging can help us understand biomolecule distribution, thus providing insight into their function. Alkyne tags, being small, can be used for modification to generate mimics that have a close resemblance to the original molecule, with little perturbation of the activity. Many of the molecules highlighted in previous sections, including EdU, AltQ, and alkyne-tagged palmitic acid, are biomolecule mimics. These and other alkyne-tagged mimics are used to study the uptake and distribution of biomolecules and bioactive molecules using ATRI (Table 1).

**Table 1:** Structures of biomolecules and bioactive molecules and their alkyne-tagged mimics discussed in the review. Modifications in the structures are highlighted in red

Biomolecule/bioactive molecule	Alkyne tagged mimic	Ref.
Deoxyribonucleoside and ribonucleoside		
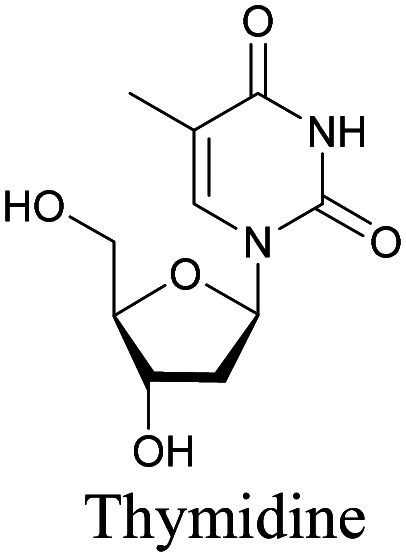	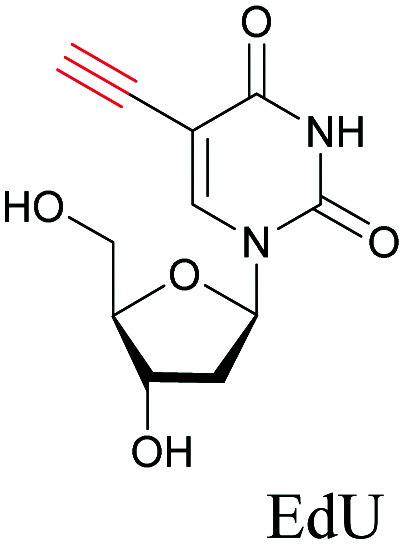	68, 89 and 100
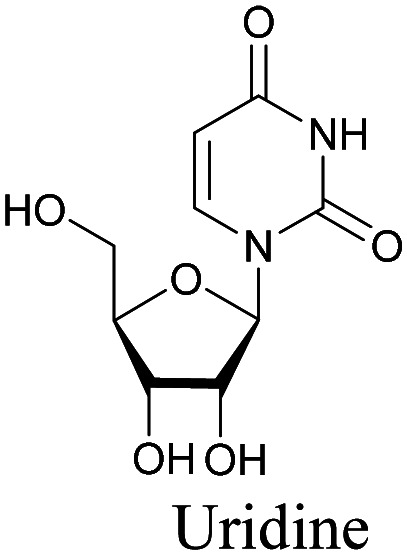	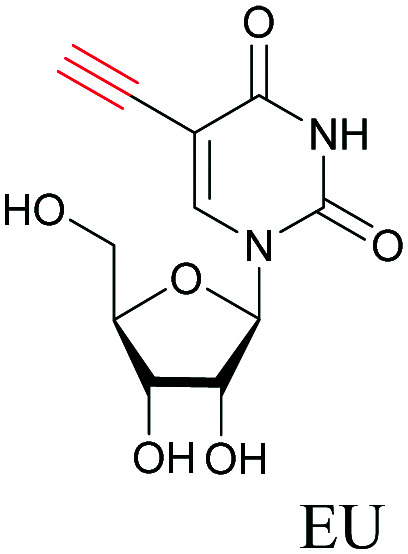	82 and 89

Lipids		
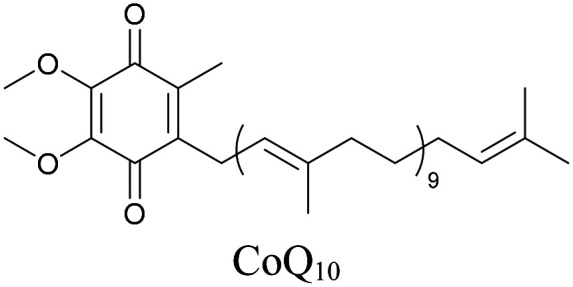	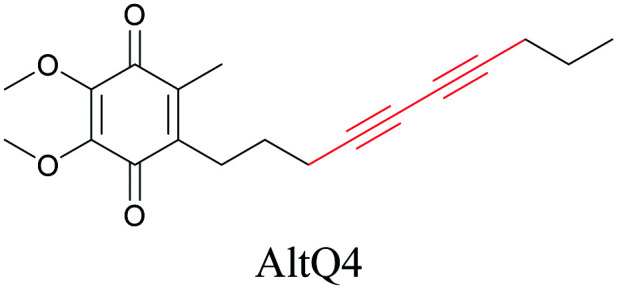	92
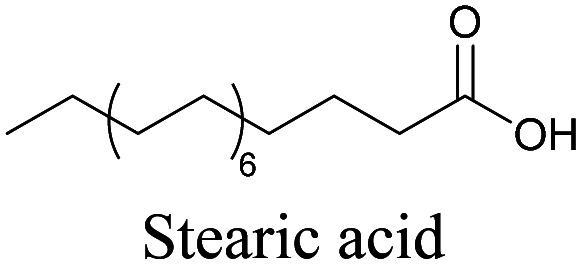	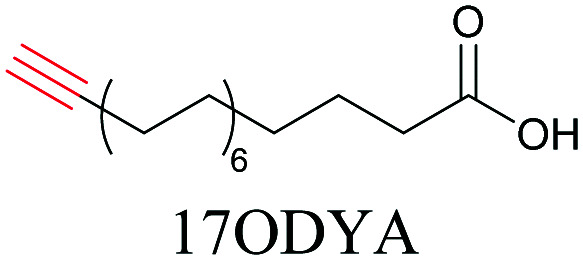	69, 89 and 100
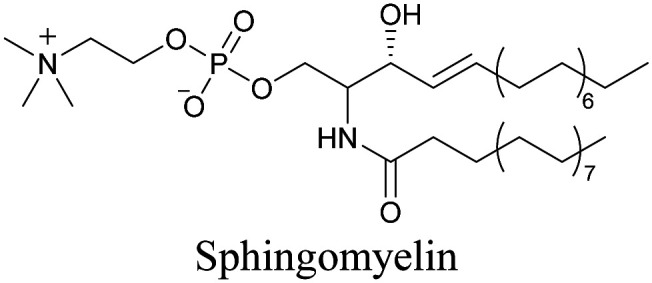	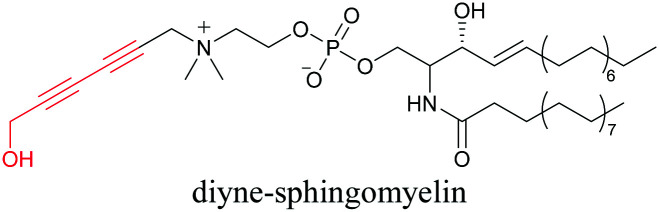	103 and 104
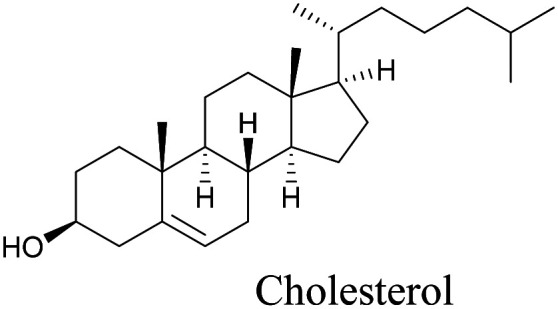	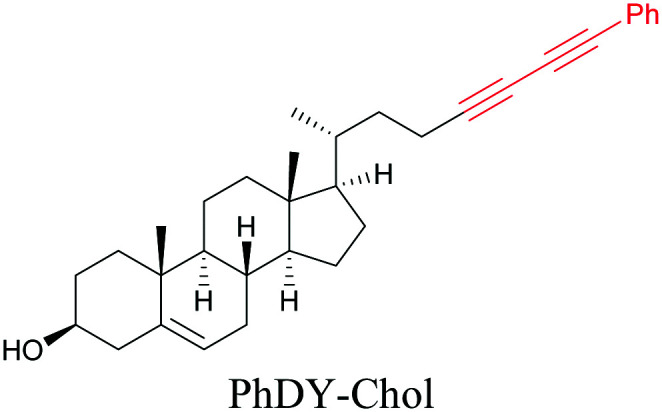	101
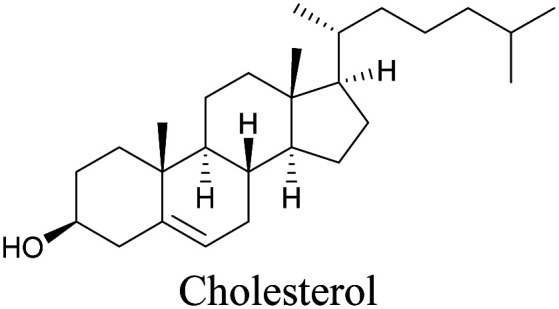	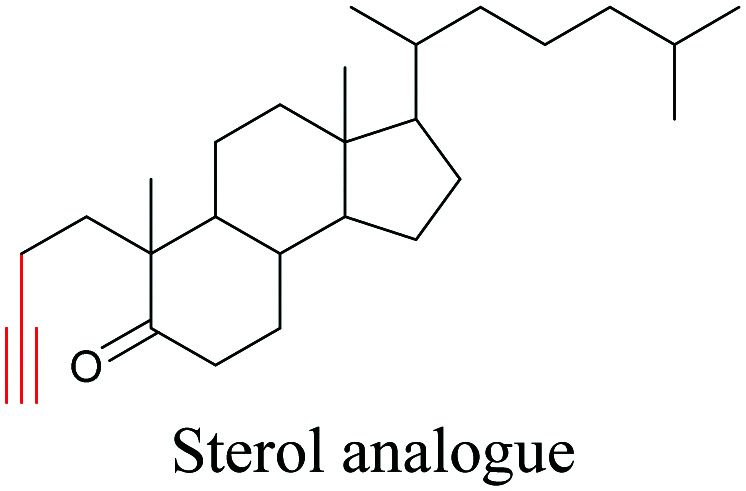	102
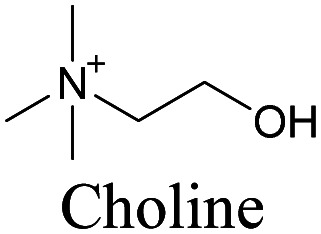	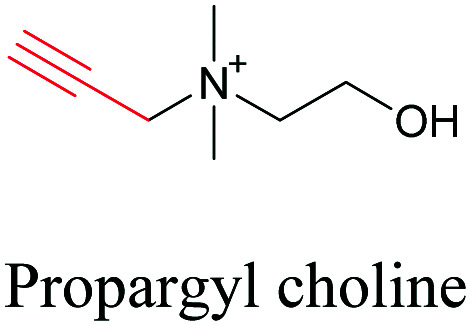	89 and 100

Sugars		
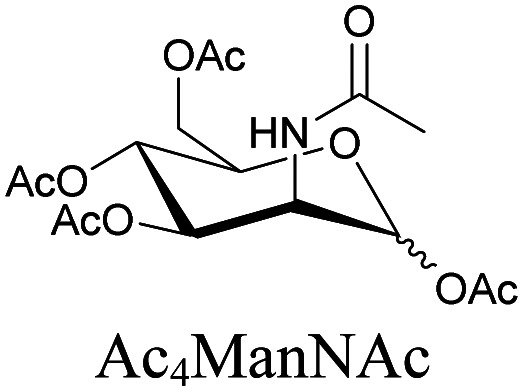	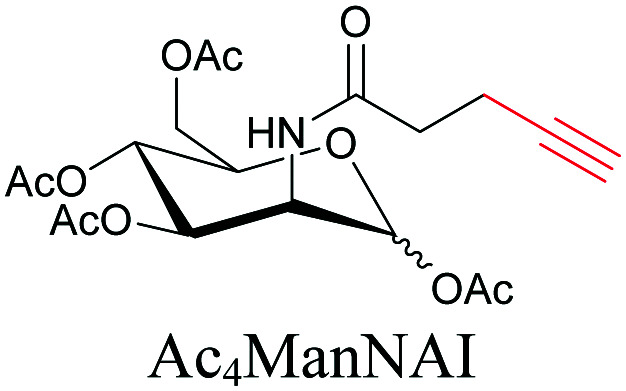	100
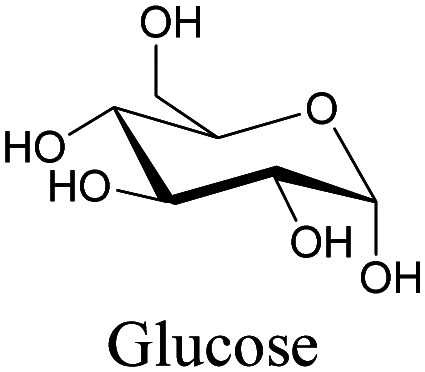	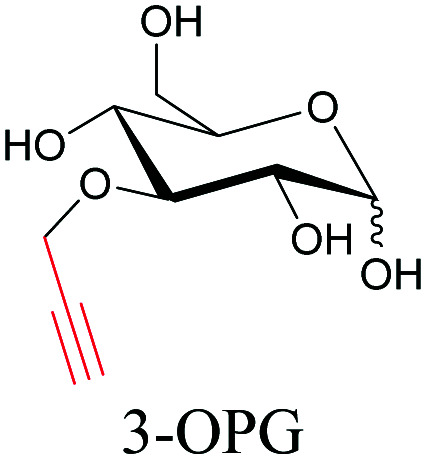	106
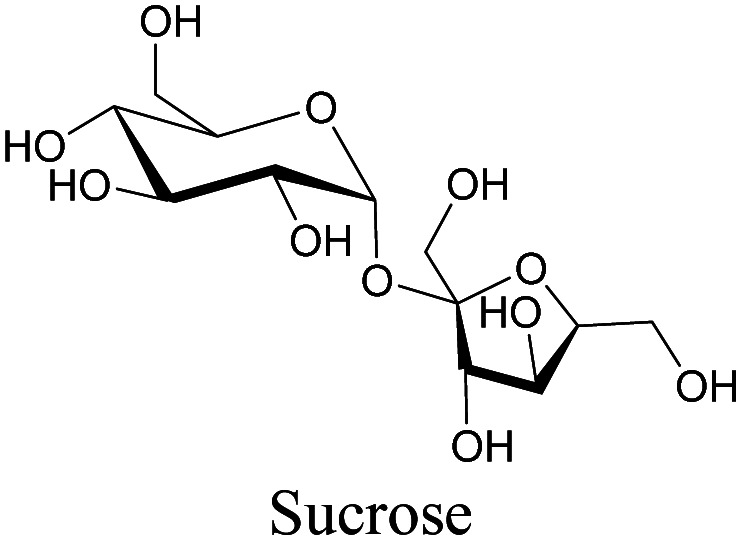	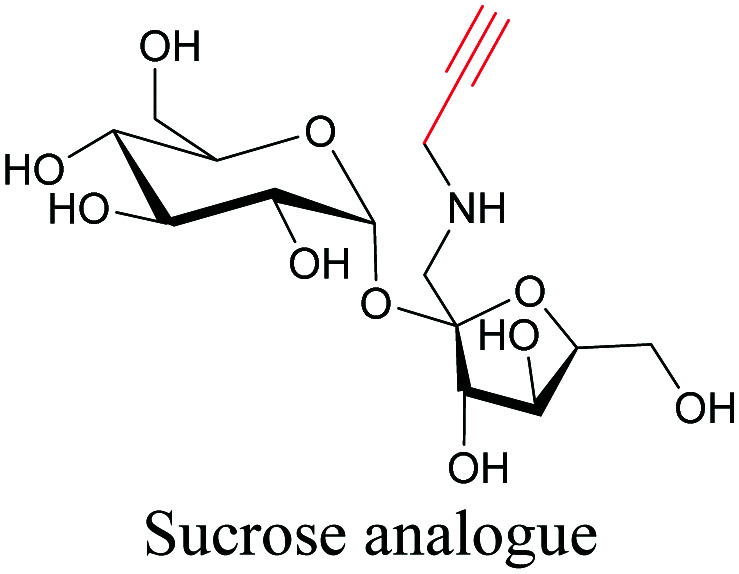	108

Amino acids		
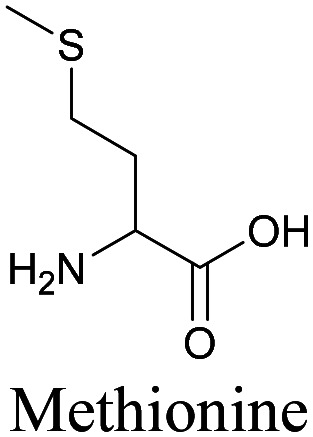	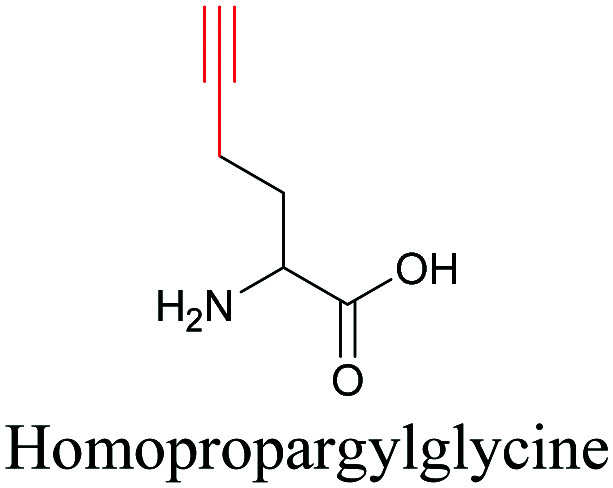	89 and 100

Natural product		
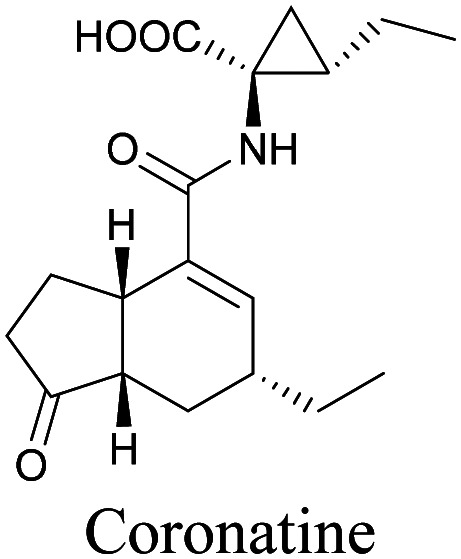	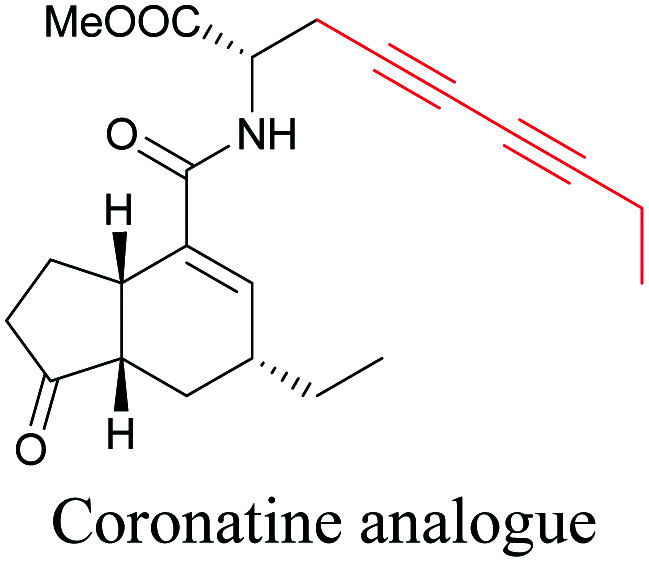	110

Bioactive molecules		
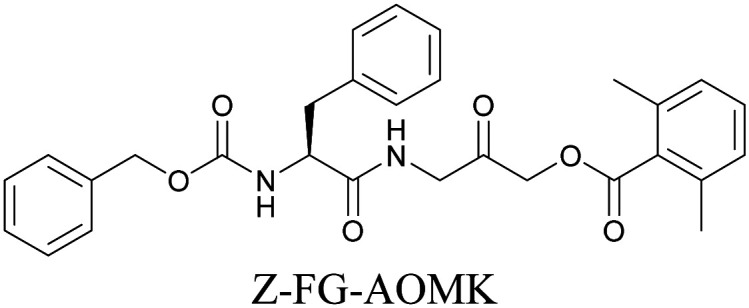	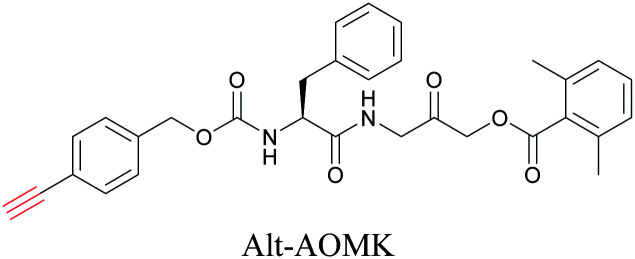	90 and 91
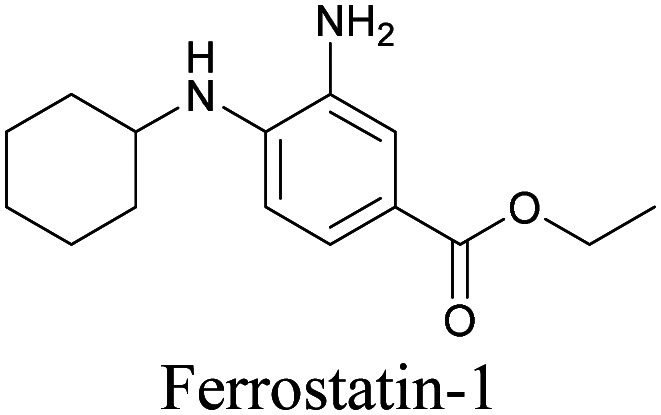	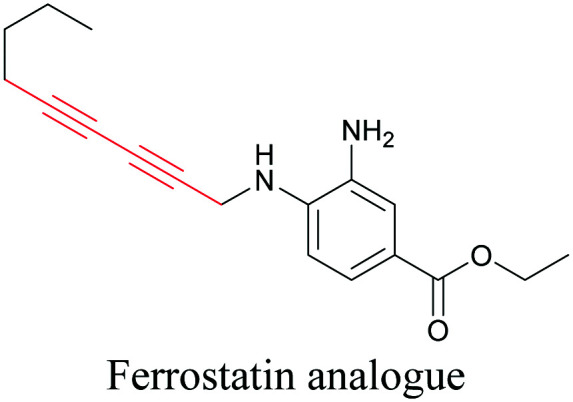	112
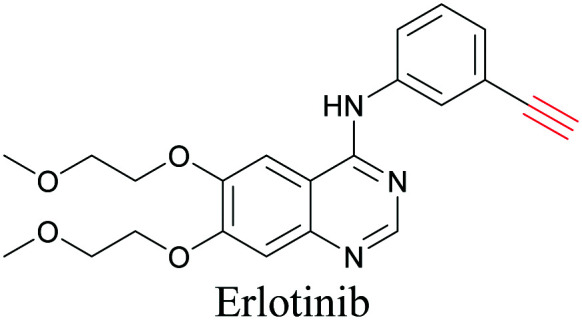	NA	113
Bioactive molecules		
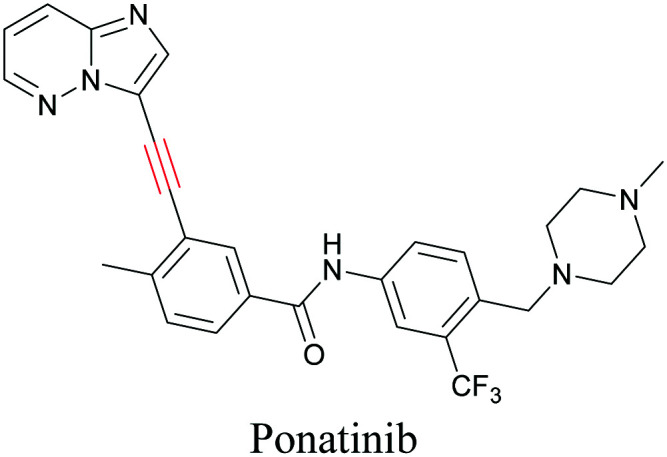	NA	114
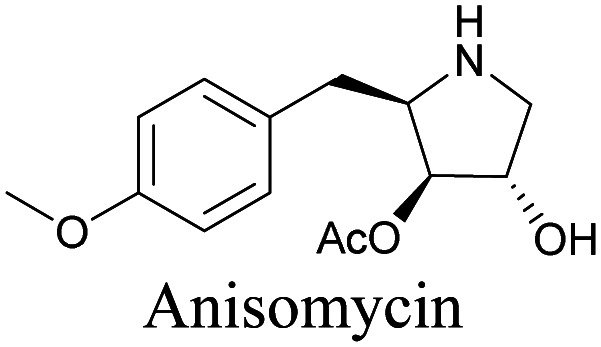	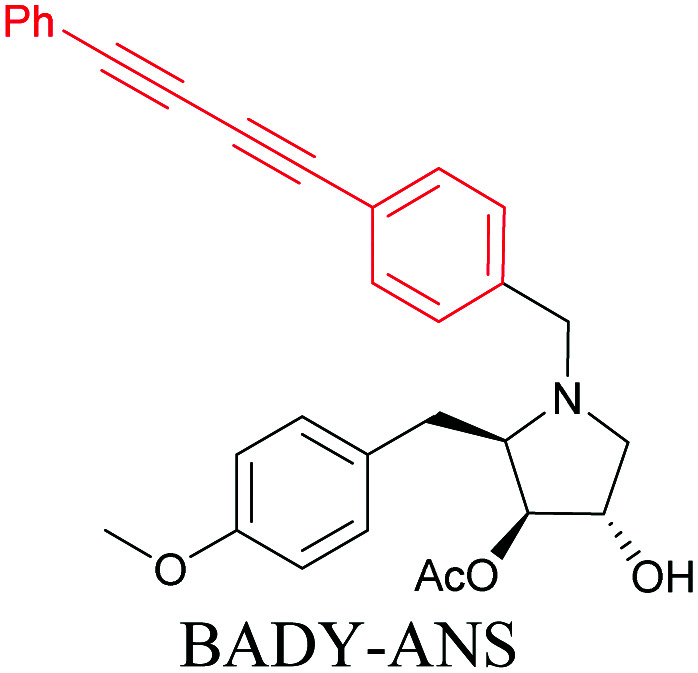	115
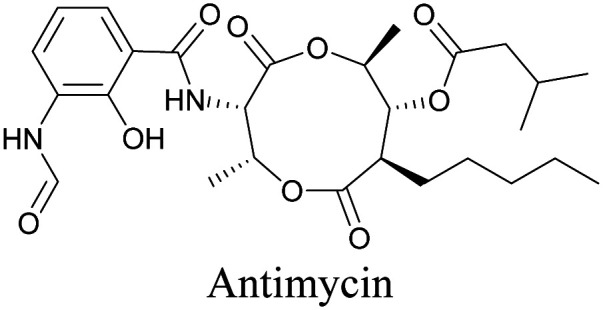	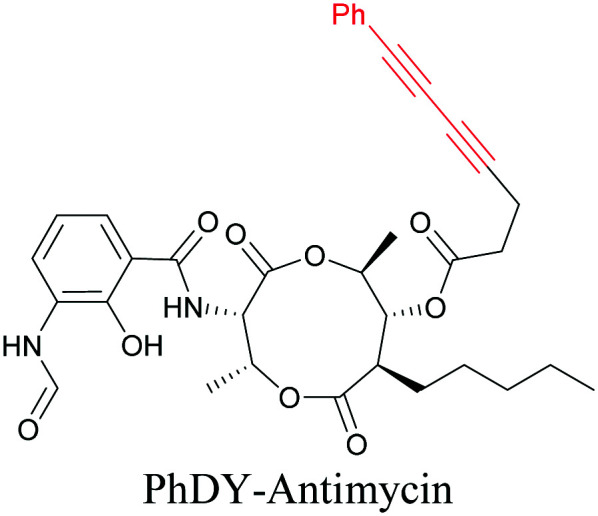	116
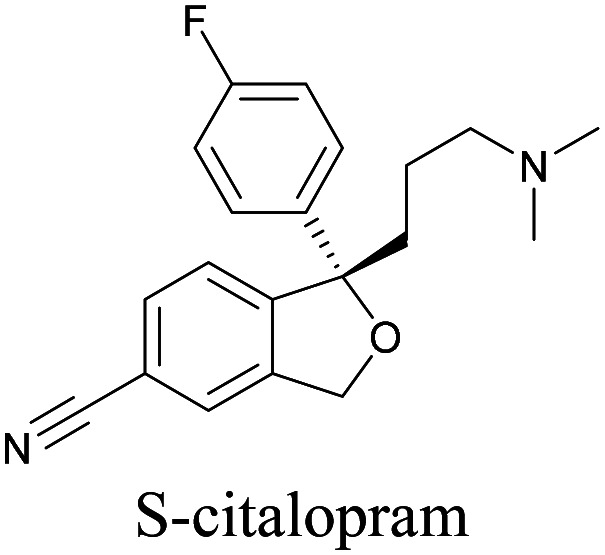	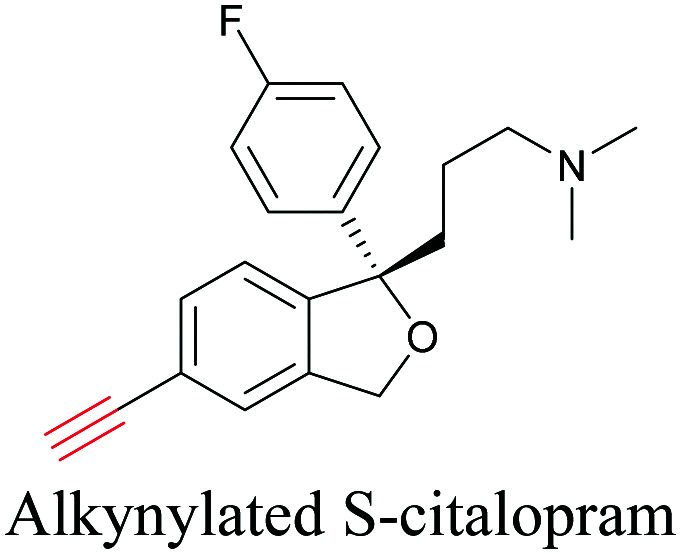	118

Similar to EdU, EU is a ribonucleoside mimic, and was used to visualize RNA metabolism using SRS. SRS imaging employing EdU and EU was used to compare DNA and RNA dynamics, and it was demonstrated that RNA turnover is much faster than DNA dynamics.^82^

By using alkyne-conjugated amino acids, biomacromolecules like proteins can be studied with ATRI. Hong *et al.* used homopropargylglycine, which is recognized and incorporated as methionine by cells, to visualize newly synthesized protein using SRS.^100^

Sugars are another important class of biomolecules. Alkyne-modified glucose and glycans have been utilized for click-based imaging, but these molecules can be visualized by ATRI without any further modifications or reactions. Hong *et al.* visualized the distribution of Ac_4_ManNAI at 2120 cm^−1^ using SRS.^100^ Min and coworkers synthesized propargyl-modified glucose (3-OPG) and examined its uptake in HeLa cells. By using various inhibitors and stimulators of glucose uptake, they revealed the quantitative variation in 3-OPG uptake. They also monitored differential uptake in tumor xenografts and mouse brain tissues.^106^ The same group synthesized 3-OPG isotopically edited with ^13^C, and performed ratiometric imaging with D7-glucose in MCF-7 cells and in live mouse brain tissues.^107^ ATRI can be applied even in plant cells. For example, de Moliner *et al.* reported real-time visualization of sucrose uptake in plant cells using an alkyne-tagged sucrose.^108^

Coronatine, a bacterial virulence factor, resembles a plant hormone and can thus hijack the plant system.^109^ Unfortunately, fluorescence-labelled coronatine loses its activity, but alkyne modification does not affect the activity. By using alkyne-tagged coronatine, Ueda *et al.* have shown that coronatine localizes in endoplasmic reticulum, but not in the nucleus, where a co-receptor for the hormone is present.^110,111^ Such studies give us helpful information to decipher the mechanism of action, as well as possible targets for intervention.

ATRI has also been used to examine the distributions of various bioactive molecules and to elucidate their mechanisms of action. Ando *et al.* reported an alkyne-tagged cathepsin B selective inhibitor Z-FG-AOMK called Alt-AOMK. The IC_50_ for cathepsin-B inhibition was unchanged.^90^ Koike *et al.* used time-lapse 3D SERS imaging to quantitatively monitor Alt-AOMK uptake by J774A.1 macrophages.^91^ Gaschler *et al.* designed a ferrostatin-1 analog with a diyne. Diyne-modified ferrostatin was reported to show similar potency. By means of SRS imaging they identified ferrostatin accumulation in lysosomes, endoplasmic reticulum, and mitochondria.^112^ Although alkyne has been used as external tags in above examples inherent alkynes in the molecules can also be used to perform label-free ATRI. El-Mashtoly *et al.* have studied subcellular distribution and metabolism of erlotinib in colon cancer cells using label-free ATRI.^113^ By using the inherent alkyne in the chronic myeloid leukaemia drug ponatinib for SRS imaging, Sepp *et al.* monitored the changes in drug uptake and sequestration during the development of drug resistance.^114^

Anisomycin, a protein synthesis inhibitor has been conjugated to BADY and its cellular uptake and distribution were imaged using SRS at 2219 cm^−1^.^115^ Antimycin is a class of natural product with potent anticancer activity. The distributions of 9-membered antimycin and 15-membered neoantimycin in HeLa and MCF-7 cells were studied using a phenyldiyne (PhDY) tag. Seidel *et al.* observed that PhDY-tagged antimycin is mainly colocalized with ER and PhDY-tagged neoantimycin is distributed throughout the cytoplasm, indicating they have different mechanisms of action.^116^

Thus, ATRI can play a key role in visualization of the uptake, storage, and distribution pattern of drugs, pointing towards possible mechanistic pathways.

### 2.4 ATRI in tissues and whole animal models

Raman imaging is also available for studies at the tissue and organism level, with particular applications in biomedical imaging such as disease diagnosis. Even though ATRI is in the early phase of its biological applications, it has already been applied for tissue and organism imaging.

**Fig. 5:**
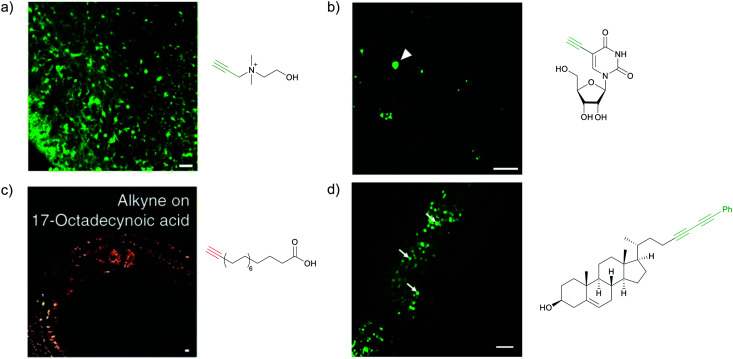
Alkyne-tag SRS imaging of (a) propargyl choline in rat hippocampal tissue at 2140 cm^−1^. Scale bar: 40 μm and (b) EU in rat hippocampal tissues at 2123 cm^−1^. White arrowhead: newly synthesized RNA in nucleoli. Scale bar: 10 μm. Reprinted from ref. 82. Alkyne tag imaging of (c) 17-ODYA in *C. elegans* at 2125 cm^−1^. Scale bar: 10 μm. Reprinted from ref. 89 by permission from Springer Nature Copyright©2014 and (d) PhDY-Chol imaging in *C. elegans* at 2254 cm^−1^. White arrows: PhDY-Chol rich region in intestine of *C. elegans*. Scale bar: 10 μm. Reprinted from ref. 101.

Hu *et al.* reported imaging of alkyne-tagged small molecules along with deuterated molecules in live rat hippocampal tissue. They were able to successfully employ these molecules for imaging to observe newly synthesized proteins and lipids, and also to identify metabolic activity changes in specific regions of hippocampal tissue in a traumatic brain injury model (Fig. 5a and b).^82^

An alkyne-based ratiometric SERS probe was developed and applied to monitor carbon monoxide in *ex vivo* tissue samples by Qin *et al.*^117^ Recently, Tanuma *et al.* used a mimic of the antidepressant *S*-citalopram (*S*-Cit) in which a nitrile is substituted for alkyne to visualize the distribution in coronal mouse brain sections with SERS.^118^ The *in silico*-predicted binding of alkyne-tagged *S*-Cit (Alk-*S*-Cit) to serotonin transporter was similar to that of *S*-Cit. Replacing nitrile with alkyne for SERS imaging did not affect brain transitivity or serotonin uptake inhibition by Alk-*S*-Cit.^118^ Such strategies can help visualize the behavior of drug molecules in the original complex tissue environment, rather than just in a single cell line. While information obtained from cell lines and tissues gives great detail about the specific activity of molecules, in some cases the *in vivo* behavior of these molecules may be different from what we see in cell lines. In such cases, it is important to understand the uptake and distribution at the organism level. Albeit a new technology, Raman imaging has been already tested for *in vivo* imaging in small animal models such as *C. elegans*. Wei *et al.* visualized 17-ODYA in *C. elegans* using SRS (Fig. 5c).^89^ Alkyne-tagged cholesterol molecules were imaged in CHO cells and *C. elegans* by Cheng and coworkers. In live *C. elegans*, cholesterol storage was observed in lysosome-related organelles rather than in lipid droplets (Fig. 5d).^101^ Li *et al.* used alkyne-tagged and deuterated fatty acids to quantitatively monitor fatty acid storage and distribution in lipid droplets of fed and starved adult *C. elegans*.^119^

Alkyne tagged polymeric and SERS nanoparticles have been used for cellular and tissue imaging.^120,121^ Recently such nanoparticles have also been used for imaging in whole animal models. Jin *et al.* developed polymeric nanoparticles with different Raman tags, including alkyne, for tumor detection. Using various targeting moieties, they were able to detect tumors and other organs in live mice using spontaneous Raman imaging.^122^ Wang *et al.* used alkyne-tagged Au nanoparticles for SERS mapping of tumors in living mice. They were also able to use these nanoparticles for photothermal therapy in mice, thus making alkyne-tagged Au nanoparticles as a theranostic agent.^123^

The examples described above indicate that ATRI is a promising methodology to monitor drug distribution and metabolism at the tissue and organism level in animals.

## 3. Conclusion and outlook

The work curated in this review article is drawn from the last few years. Until now, most studies have been focused on establishing and validating the technique, and there is huge potential yet to be exploited. Taking a cue from the alkyne-tagged molecules so far used for ATRI (Table 1), challenging small molecules of interest might also be tailored with alkynes for visualization in biological samples with ATRI.

Combining ATRI with other techniques may also extend its usefulness. By combining alkyne tagging with SERS screening, we have developed alkyne tag Raman screening, ATRaS. With this technique we were able to identify small-molecule binding pockets of proteins.^90^ By applying a genetic modification strategy, Zhang *et al.* introduced an unnatural amino acid bearing an alkyne into proteins and visualized histone3.3. Instead of attaching a fluorophore *via* click chemistry, they attached bisarylbutadiyne for imaging using ATRI, and successfully visualized the distribution of Sec61β and Htt74Q proteins in HeLa cells.^124^

Alkyne moieties can be used as reporters, not only as tags. Small structural modifications can also give us distinguishable changes in Raman signals. This opens up the possibility of developing ‘turn-on’ or ratiometric sensors for Raman imaging. Zeng *et al.* have developed this strategy, using azide-substituted aromatic diyne to visualize mitochondrial H_2_S. Azide in the presence of H_2_S was reduced to amine, which was detected based on the shift in the diyne Raman signal from 2223 cm^−1^ to 2214 cm^−1^.^125^ Wilson *et al.* synthesized a series of diynes with a range of p*K*_a_ values. They used these diynes to measure pH in the range from 2 to 10, based on the Raman shift. They used ratiometric sensing to determine the pH of PC3 cells and also to monitor the change of pH upon treatment with etoposide, an apoptosis inducer.^126^ Changes in the SERS enhancement of alkynes have also been used to monitor the cellular environment.^127–129^ Currently we are working to develop a strategy of structural-change-based ratiometric ATRI to detect reactive biological molecules. One of the recent developments is photoswitchable Raman probes containing alkyne as reporter moiety. Ao *et al.* have developed photoswitchable molecules with alkyne whose Raman signal at 2194 cm^−1^ can be turned on using UV and turned off using visible light. Using SRS imaging, they demonstrated the photoswitching ability in live HeLa cells and also visualized mitochondria diffusion using pulse-chase experiments.^130^ Similar to switchable nitrile probes, photoswitchable alkyne probes also have potential application toward super-resolution SRS imaging in the cell silent region. Raman imaging is already used in clinical diagnostics and is being translated from bench to bedside.^131–133^ When appropriate reporters are developed, ATRI can play an expanding role in future medical diagnosis. ATRI is still at its infancy, but we believe it could become an essential tool for life sciences research in the near future.

## Conflicts of interest

There are no conflicts to declare.

## Supplementary Material
